# Targeting mTORC1 to promote ferroptosis and apoptosis in endometrial cancer with PI3K-Akt-mTOR pathway mutation

**DOI:** 10.70401/fos.2025.0005

**Published:** 2025-11-26

**Authors:** Yingying Hu, Pei Liu, Neal Rosen, Xuejun Jiang

**Affiliations:** 1 Cell Biology Program, Memorial Sloan Kettering Cancer Center, New York, NY 10065, USA.; 2 Tri-Institutional PhD Program in Chemical Biology, Memorial Sloan Kettering Cancer Center, New York, NY 10065, USA.; 3 Program in Molecular Pharmacology, Memorial Sloan Kettering Cancer Center, New York, NY 10065, USA.

**Keywords:** Endometrial cancer, PI3K-Akt-mTOR pathway, ferroptosis, apoptosis, mTORC1, combination therapy

## Abstract

**Aims:**

Endometrial cancer (EC) is often driven by hyperactivation of the PI3K-Akt-mTOR (PAM) pathway due to mutations in PTEN and/or PI3K genes. While mechanistic target of rapamycin complex 1 (mTORC1) inhibitors show limited efficacy as single agents in EC, previous studies suggest that they may sensitize the PAM-mutant cancer cells to ferroptosis, a regulated form of necrosis dependent on iron-catalyzed lipid peroxidation. We investigated whether combining mTORC1 inhibition with ferroptosis induction could overcome resistance mechanisms and improve therapeutic outcomes in EC.

**Methods:**

We evaluated the effect of catalytic, allosteric, and bi-steric mTORC1 inhibition on ferroptosis sensitivity in EC cell lines with different PAM pathway mutational statuses. *In vivo* efficacy of the combinational treatment was tested in MFE296 xenograft models.

**Results:**

The catalytic and bi-steric mTORC1 inhibitor RMC-6272 sensitized PAM pathway-activated EC cells to ferroptosis induced by GPX4 inhibition, while EC cells without PAM pathway activation were intrinsically sensitive to ferroptosis. Further, mTORC1 inhibition also induced apoptosis in PAM pathway-activated EC cells, indicating a multi-modal cell death response. *In vivo*, combination treatment with RMC-6272 and the GPX4 inhibitor JKE-1674 significantly suppressed xenograft growth, with evidence of both ferroptosis and apoptosis in tumors.

**Conclusion:**

Our study highlights the therapeutic potential of dual targeting of mTORC1 and ferroptosis to trigger multi-modal cell death in PAM pathway-activated EC, with broader implications for other cancers exhibiting mTORC1 hyperactivation.

## 1. Introduction

Endometrial cancer (EC) is the most common malignancy of female genital tract and is the fourth most common cancer among women in the United States as in 2025^[1]^, and the sixth most common worldwide^[2]^. Approximately two-thirds of EC cases are diagnosed at an early stage, largely because abnormal vaginal bleeding is an early symptom^[3]^. Nevertheless, the EC mortality rate has increased rapidly over the past 50 years in the United States, driven by both increasing incidence and a lack of substantial improvement in survival^[4]^. Current standard-of-care treatment for EC typically involves surgery and hormone therapies, particularly for early stage EC. However, aggressive or non-hormone-dependent EC subtypes often have poorer prognosis^[5]^, highlighting the need for new therapeutic approaches. Several immunotherapies, targeted therapies, and their combinational therapies are under active development^[6,7]^.

**Figure 1.**
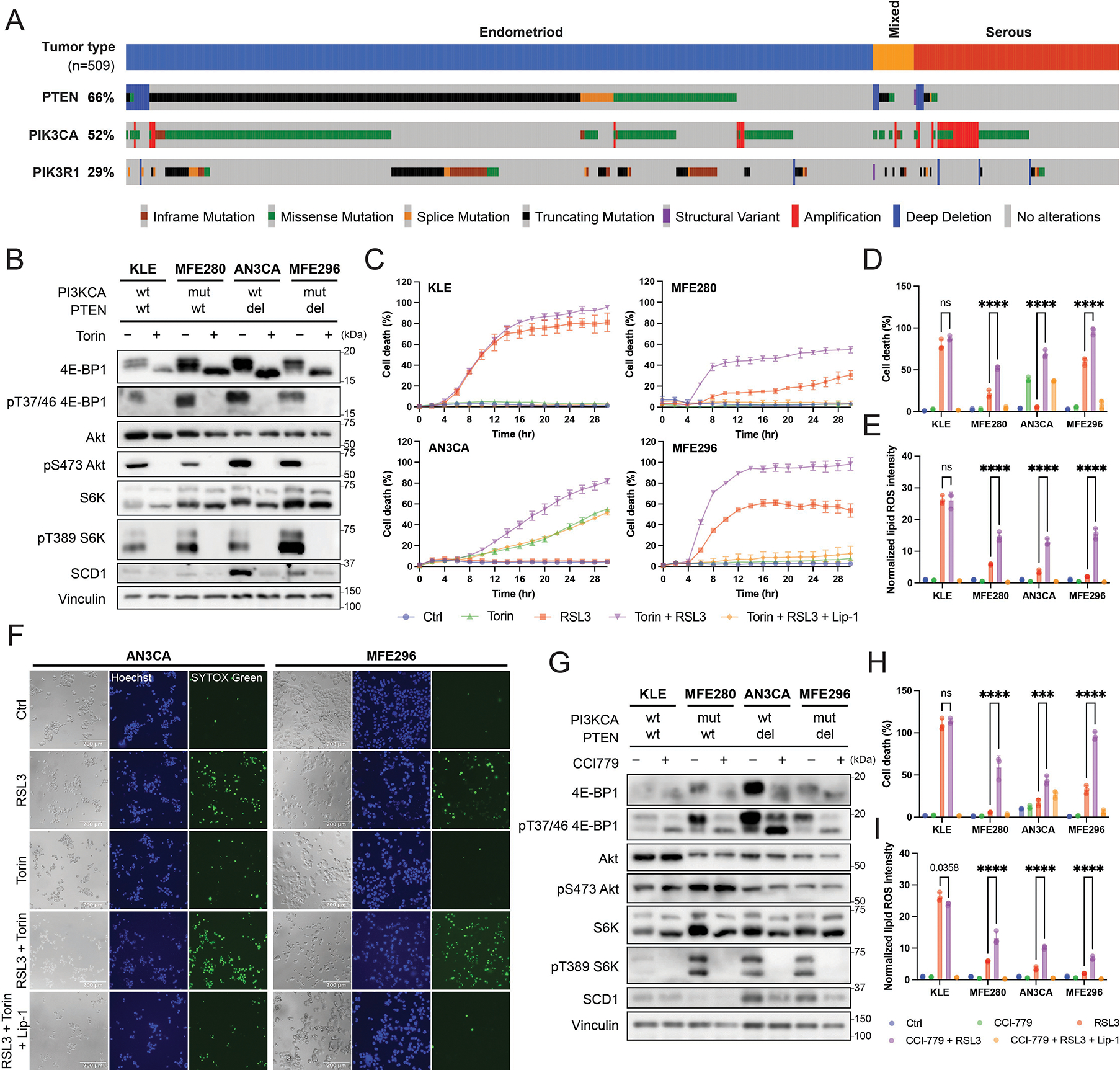
mTORC1 inhibition sensitizes EC cells towards ferroptosis. (A) ECs exhibit high mutation rate in the PAM pathway activating mutants. Figure is adapted from the cBioPortal for Cancer Genomics, data obtained from TCGA PanCancer Atlas^[8–10]^; (B) Torin treatment of 8 h in EC cell lines inhibits mTORC1 and mTORC2; (C) Cell death curve after Torin and RSL3 treatment; (D) Cell death percentage at 24 h post Torin and RSL3 treatment; (E) Normalized lipid ROS intensity of EC cell lines under Torin and RSL3 treatment at 4 h; (F) Representative image of cell death after 24 h of treatment with Torin and RSL3 on AN3CA and MFE296 cells; (G) CCI-779 treatment of 8 h in EC cell lines inhibits mTORC1; (H) Cell death at 24 h following 24 h pretreatment with CCI-779 and subsequent RSL3 treatment on EC cell lines; (I) Normalized lipid ROS intensity of EC cell lines at 4 h following 24 h pretreatment with CCI-779 and subsequent RSL3 treatment. Torin, 1 μM; CCI-779, 2 μM; RSL3, 50 nM; Lip-1, 2 μM. Data are presented as mean ± SD, *n* = 3 (C-E, H-I) biologically independent samples. Statistical analysis was performed using two-way ANOVA (D-E, H-I). In all panels with combination treatment, when CCI-779 was used, cells were pretreated with CCI-779 for 24 hr before the indicated combination treatment. mTORC1: mechanistic target of rapamycin complex 1; EC: endometrial cancer; PAM: PI3K-Akt-mTOR; ROS: reactive oxygen species; ANOVA: analysis of variance; SD: standard deviation.

According to genomic profiling by The Cancer Genome Atlas (TCGA)^[8–10]^, phosphatase and tensin homolog (PTEN) and PIK3CA, both key components of the PI3K-AKT-mTOR (PAM) pathway, are among the most frequently mutated genes in EC. Notably, PTEN and PI3K mutations co-occur in around 60% of EC cases, and over 90% of ECs harbor at least one alteration in the pathway^[11–13]^, making this pathway an attractive therapeutic target (Figure 1A). The basis of this unusually high co-mutation rate, considering both having similar function of activating the pathway, remains unclear. Several PAM pathway-targeted therapeutic agents, such as mTORC1 inhibitors Afinitor (Everolimus) and CCI-779 (Temsirolimus), have been approved by Food and Drug Administration (FDA) for EC treatment^[14]^. However, response rates to single agent mechanistic target of rapamycin complex 1 (mTORC1) inhibition vary, showing benefit in certain EC subgroups but limited efficacy in many others^[15–22]^. Furthermore, PAM pathway-targeted therapies are often met with toxicity issues, motivating exploration of combinational therapies to enable lower dosing while maintaining or enhancing efficacy.

The PAM pathway coordinates various signaling pathways and environmental inputs to regulate eukaryotic cell growth, metabolism, and survival^[23]^. In cancer, its oncogenic activation drives transcriptional and translational reprogramming, tumor progression, and immune invasion^[24]^. mTOR functions in two distinct complexes: mTORC1 and mTORC2, which have different substrates and co-factors, allowing independent pathway regulation. While mTORC1 impacts cancer cell invasion, metastasis, protein synthesis, and autophagy via phosphorylation of its various substrates^[25]^, mTORC2 exerts a strong effect on cell growth and survival mainly via phosphorylation of Akt^[26,27]^. Catalytic mechanistic target of rapamycin (mTOR) inhibitors, which block both mTORC1 and mTORC2, often demonstrate stronger tumor cell killing but also higher toxicity and adverse effects^[27]^. In contrast, mTORC1-specific inhibitors, specifically rapalogs (Rapamycin analogs), tend to stabilize rather than regress tumor.

We recently discovered a mechanistic link between the PAM pathway and ferroptosis^[28]^, a regulated form of cell death driven by lipid peroxide accumulation^[29,30]^. Oncogenic activation of the PAM pathway leads to mTORC1 activation, which in turn stimulates the transcription factor and master regulator of lipid metabolism, sterol regulatory element-binding transcription factor 1 (SREBP1)^[28]^. SREBP1 upregulates stearoyl-CoA desaturase-1 (SCD1), an enzyme catalyzing the conversion of saturated fatty acids (SFAs) into monounsaturated fatty acids (MUFAs), which are inhibitors of ferroptosis^[31,32]^.

Based on these earlier findings, we hypothesized that combining PAM pathway inhibition with ferroptosis induction could be an effective strategy for EC treatment, particularly for those with co-mutations in the pathway. Here, we evaluated this combination treatment in EC cell lines with different PAM pathway mutation statuses. We demonstrate that the bi-steric inhibitor RMC-6272 sensitizes PAM pathway-activated EC cell lines to ferroptosis at low concentrations, potentially reducing toxicity and adverse side effect associated with mTORC2 inhibition, such as hyperglycemia^[33]^. Importantly, RMC-6272 also induced apoptosis in those PAM pathway-activated EC cell lines. Together, the combination of mTORC1 inhibition with ferroptosis activation engages multiple cell death pathways and represents a promising therapeutic option for EC.

## 2. Methods

### 2.1 Chemicals

Torin-1 (Selleck Chemicals S2827) were purchased from Selleck Chemicals. (1S,3R)-RSL3 (Cayman Chemicals 19288), Liproxstatin-1 (Cayman Chemicals 17730), and IM-54 (Cayman Chemicals 13323) were purchased from Cayman Chemicals. CCI-779 (Temsirolimus, MedChem Express HY-50910), RMC-6272 (RM-006, MedChem Express HY-134904), A939572 (MedChem Express HY-50709), Necrostatin-1 (MedChem Express HY-15760), Z-VAD-FMK (MedChem Express HY-16658), Solutol HS-15 (MedChem Express HY-Y1893), and JKE-1674 (MedChem Express HY-138153) were purchased from MedChem Express. Transcutol (Diethylene glycol monoethyl ether, Sigma-Aldrich 537616) was purchased from Millipore Sigma.

### 2.2 Cell culture

KLE, AN3CA, MFE280, and MFE296 cell lines were a gift from the Neal Rosen lab. KLE, AN3CA, MFE280, MFE296 cells, and their derived cell lines were cultured in DMEM:F12 (provided by the Media Preparation Core Facility of MSKCC) with 10% Fetal bovine serum (Gibco), 2 mM L-glutamine (provided by the Media Preparation Core Facility of MSKCC), and 100 U/mL Penecillin/Streptomycin (Gibco) at 37 °C and 5% CO_2_. All cell lines were tested for mycoplasma contamination.

### 2.3 Generation of Bcl-xL stable expression cells

Bcl-xL overexpression retroviral plasmid were obtained as described^[34]^. Retroviruses were produced by co-transfecting the retroviral plasmid, packaging plasmid (gag/pol Addgene#14887), and pCMV-VSV-G plasmid (Addgene #8454) into HEK293T by Lipofectamine 3000 reagent (Invitrogen^™^). The supernatants containing infectious particles were collected 48 h post transfection and passed through a 0.45-μM filter. To generate Bcl-xL overexpression cell line, the indicated cells were infected with retroviruses and polybrene (Sigma Aldrich TR-1003) and selected by 2 μg/mL puromycin.

### 2.4 Cell death assay

Cells were seeded at an appropriate cell density and cultured in normal condition in 24-well plates overnight. Cells were then stained with Hoechst 33342 (Invitrogen^™^ H3570) at 1 μg/mL for 20 min at 37 °C to visualize individual nuclei. Medium containing Hoechst 33342 was removed, and cells were treated with the indicated treatments as described in individual experiments with SYTOX^™^ Green Nucleic Acid Stain 50 nM (Invitrogen^™^ S7020) to monitor cell death with the BioTek BioSpa live cell analysis system. Alternatively, images were taken by a Nikon Eclipse Ti2 microscope.

### 2.5 Measurement of lipid ROS

Cells were seeded at an appropriate cell density and cultured under normal conditions in 24-well plates. Torin, CCI-779, and RMC-6272 were treated at corresponding concentrations overnight. Then, the cells were treated with RSL3 for 3 hours, followed by staining with BODIPY^™^ 581/591 C11 (Invitrogen^™^ D3861) at 5 μM for 30 min at 37 °C. The cells were then washed twice with PBS and trypsinized for flow cytometry analysis with 0.1 μg/mL DAPI (Invitrogen^™^ D1306).

### 2.6 Immunoblotting

For cultured cell lines, cells were harvested and lysed in RIPA buffer containing 0.5 μM Leupeptin, 2 μM Aprotinin, and 1 μM Pepstatin A. For xenograft tissues, cells were homogenized in RIPA buffer with protease inhibitor. Cell debris was removed by centrifugation at 14,000 rpm for 10 min, and the supernatant were collected. Protein concentration was determined by Protein Assay Dye Reagent Concentrate (Bio-Rad 5000006). 4x Laemmli buffer was added to the cell lysate at appropriate concentration, and the samples were boiled at 95 °C for 5 min. Cell lysates were then resolved on SDS/PAGE gels and transferred to a nitrocellulose membrane. The membranes were blocked in 5% milk (RPI M17200) at room temperature for 30 min, then incubated with primary antibodies at 4 °C overnight. After three washes, the membranes were incubated with goat anti-mouse HRP-conjugated antibody (Invitrogen^™^ 31430) or goat anti-rabbit HRP-conjugated antibody (Invitrogen^™^ 31460) at room temperature for 30 min, followed by three washes. The membranes were then incubated with Clarity Western ECL Substrate (Bio-Rad 1705060) for 5 min for chemiluminescence and visualized by Amersham Imager 600 (GE Healthcare Life Sciences).

The following primary antibodies were used: 4E-BP1 Antibody (Cell Signaling Technology 9452S), 1:1,000; Phospho-4E-BP1 (Thr37/46) (236B4) Rabbit mAb (Cell Signal Technology 2855S); Akt (pan) (40D4) Mouse mAb (Cell Signaling Techonology 2920S), 1:1,000; Phospho-Akt (Ser473) (D9E) XP^®^ Rabbit mAb (Cell Signaling Technology 4060S), 1:1,000; p70 S6 Kinase (49D7) Rabbit mAb (Cell Signaling Technology 2709S), 1:1,000; Phospho-p70 S6 Kinase (Thr389) (108D2) Rabbit mAb (Cell Signaling Technology 9234S), 1:1,000; SCD Polyclonal Antibody (Invitrogen PA5-19682), 1:1,000; Vinculin Antibody (7F9) (Santa Cruz sc-73614), 1:1000; Anti-β-Actin antibody, Mouse monoclonal (Sigma-Aldrich A1978), 1:1,000; Purified Rabbit Anti-Bcl-x (BD Biosciences 610212), 1:1,000; Caspase-3 (D3R6Y) Rabbit mAb (Cell Signaling Technology 14220S), 1:1,000; Cleaved Caspase-3 (Asp175) (5A1E) Rabbit mAb (Cell Signaling Technology 9664S), 1:1,000; Anti-Cleaved PARP1 antibody [E51] (Abcam ab32064), 1:1,000.

### 2.7 *In vivo* mice xenograft model

MFE296 cells were implanted by injecting 6 × 10^6^ cells in 50% Matrigel (Corning CB354248) subcutaneously into the right flank of 5- to 6-wk old female NOD.CB17-Prkdc^scid^ mice (Jackson Laboratory). Tumor sizes were monitored regularly via external caliper measurements and randomized into five groups when the mean tumor volume exceeded 250 mm^3^: the vehicle control group (*n* = 6), the RMC-6272 group (*n* = 7), the JKE-1674 group (*n* = 6), the RMC-6272 + JKE-1674 group (*n* = 8), and the RMC-6272 + JKE-1674 + Liproxstatin-1 group (*n* = 5). RMC-6272 was dissolved in 5% Transcutol and 5% Solutol HS-15 in water and administered at 3 mg/kg i.p. weekly. JKE-1674 was dissolved in 10% ethanol and 90% PEG400 and administered at 25 mg/kg, p.o. every other day. Liproxstatin-1 was dissolved in 65% D5W (5% dextrose in water), 5% Tween-80, and 35% PEG-400 and administered 25 mg/kg, i.p. daily. The width (*W*) and length (*L*) of the tumor were measured every other day, and the volume (*V*) was calculated using the formula: *V = (W*^*2*^*L)/2*. The experiment ended when any tumor exceeded a volume of 2,000 mm^3^, 1.5 cm in diameter, or 10% of body weight, which was the predetermined experiment endpoint. Several mice from the RMC-6272 group (*n* = 4) and the RMC-6272 + JKE-1674 group (*n*=4) were randomly selected and continued treatment until any tumor in these mice exceeded a volume of 2,000 mm^3^, 1.5 cm in diameter, or 10% of body weight. All protocols for animal experiments were approved by the MSKCC Institutional Animal Care and Use Committee.

### 2.8 Histology and immunohistochemistry

Tumors were collected, fixed in 4% paraformaldehyde overnight, embedded in paraffin, and sectioned for immunohistochemistry and hematoxylin and eosin (H & E)-staining. Sectioned tumors were dehydrated, and antigen retrieval was performed in boiled Antigen Retrieval Buffer (EDTA buffer, pH 9.0, HelloBio HB7943) for 15 min, followed by 3% hydrogen peroxide (Fisher BP2633500) for 15 min after cooling the slides. Slides were incubated with primary antibody overnight at 4 °C. After washing, slides were incubated with SignalStain^®^ Boost IHC Detection Reagent (HRP) (Cell Signaling Technology 8114S for Rabbit or 8125P for Mouse) for 30 min. Slides were then stained with the SignalStain^®^ DAB Substrate Kit (Cell Signaling Technology 8059S) followed by Hematoxylin (Cell Signaling Technology 14166S), and imaged under a microscope.

The following primary antibodies were used: Ki-67 (D2H10) Rabbit mAb (Cell Signaling Technology 9027S), 1:400; 4-Hydroxynonenal Antibody (R&D Systems MAB3249), 1:500; Cox2 (D5H5) XP^®^ Rabbit mAb (Cell Signaling Technology 12282S), 1:500; Anti-Cleaved PARP1 antibody [E51] (Abcam ab32064), 1:100; Phospho-S6 Ribosomal Protein (Ser235/236) Antibody (Cell Signaling Technology 2211S), 1:500; Phospho-Akt (Ser473) (D9E) Rabbit Monoclonal Antibody (Cell Signaling Technology 4060S).

### 2.9 Statistical analysis

Please refer to the figure legends or the corresponding methods section for the description of sample sizes and statistical tests performed. Data are presented as mean ± SD from three or more independent experiments, or as mean ± SEM for animal experiments. Data were plotted by GraphPad Prism 10 unless otherwise specified. Differences were considered statistically significant when the *p*-value was less than 0.05; otherwise they were considered not significant (ns). *p < 0.05, **p < 0.01, ***p < 0.001, and ****p < 0.0001.

## 3. Results

### 3.1 mTOR inhibition sensitizes endometrial cancer with PAM pathway activation to ferroptosis

To assess how different types of PAM pathway activation affects the sensitivity of EC to ferroptosis, we selected four different EC cell lines with distinct PAM-pathway activation statuses: KLE, with no mutations in the pathway; MFE280, with an activating mutation in PIK3CA; AN3CA, with PTEN deletion; and MFE296, with alterations in both PIK3CA and PTEN.

We treated cells with Torin (Torin-1), a catalytic mTOR inhibitor that directly inhibits mTOR kinase activity ^[35]^. Torin inhibited the phosphorylation of T37/46 4E-BP1, S473 Akt, and T389 S6K (Figure 1B), confirming complete inhibition of both mTORC1 and mTORC2. To assess its effect on ferroptosis sensitivity, Torin treatment was combined with RSL3, a GPX4 inhibitor that induces ferroptosis (Figure 1C,D,E,F; Figure S1A). While mTOR inhibition had minimal effect on ferroptosis of KLE cells, it sensitized the other EC cell lines to RSL3-induced ferroptosis (Figure 1C,D, F) and increased lipid ROS accumulation (Figure 1E; Figure S1A). Induction of ferroptosis by IKE (Imidazole Ketone Erastin, an inhibitor of SLC7A11, the catalytic subunit of the system x_c_^−^ cystine/glutamate antiporter) was also tested, but those EC cell lines with PAM pathway activation were resistant to IKE (Figure S1B). Notably, KLE cells had low phosphorylation of S6K, as the PAM pathway in these cells is not mutated. Importantly, these PAM pathway-wildtype cells were less resistant to ferroptosis induced by both RSL3 and IKE compared with the PAM pathway-mutant cells, which is consistent with our previous findings^[28]^.

### 3.2 mTORC1 confers ferroptosis resistance in PAM pathway-activated EC cells

Among the two complexes of mTOR, mTORC1 is responsible for the activation of the SCD1/SREBP1 axis and hence the resistance towards ferroptosis^[28]^. To confirm whether this is the case in EC, we treated EC cells with CCI-779, an FDA-approved rapalog that selectively inhibits mTORC1 at an allosteric site^[36]^. CCI-779 completely inhibited phosphorylation of T389 S6K but did not affect phosphorylation of S473 Akt, confirming selective mTORC1 inhibition (Figure 1G). Partial inhibition of phosphorylated 4E-BP1 was observed, consistent with allosteric binding producing selective effects compared with catalytic inhibitors like Torin^[37]^. We pretreated EC cells with CCI-779 for 24 hours, followed by RSL3 treatment, and monitored cell death. Similar to Torin, CCI-779 had minimal effect on KLE cells, which were sensitive to RSL3 even in the absence of mTOR inhibition, but it resulted in strong sensitization of PAM-mutant EC cells to ferroptosis, as shown by both cell death (Figure 1H; Figure S1C) and lipid ROS intensity (Figure 1I; Figure S1D). These results show mTORC1 inhibition overcomes ferroptosis resistance in PAM-mutant EC cells.

### 3.3 Bi-steric mTORC1 inhibitor RMC-6272 effectively sensitizes EC to ferroptosis

Due to the toxicity and efficacy limitations of current mTOR inhibitors, several bi-steric mTORC1 inhibitors, including RMC-6272, were developed^[38,39]^. These bi-steric mTORC1-specific inhibitors combine a catalytic inhibitor and a rapalog via a chemical linker. In this way, the rapalog unit can direct the drug for mTORC1 specificity, while the catalytic inhibitor unit enhances the efficacy.

**Figure 2.**
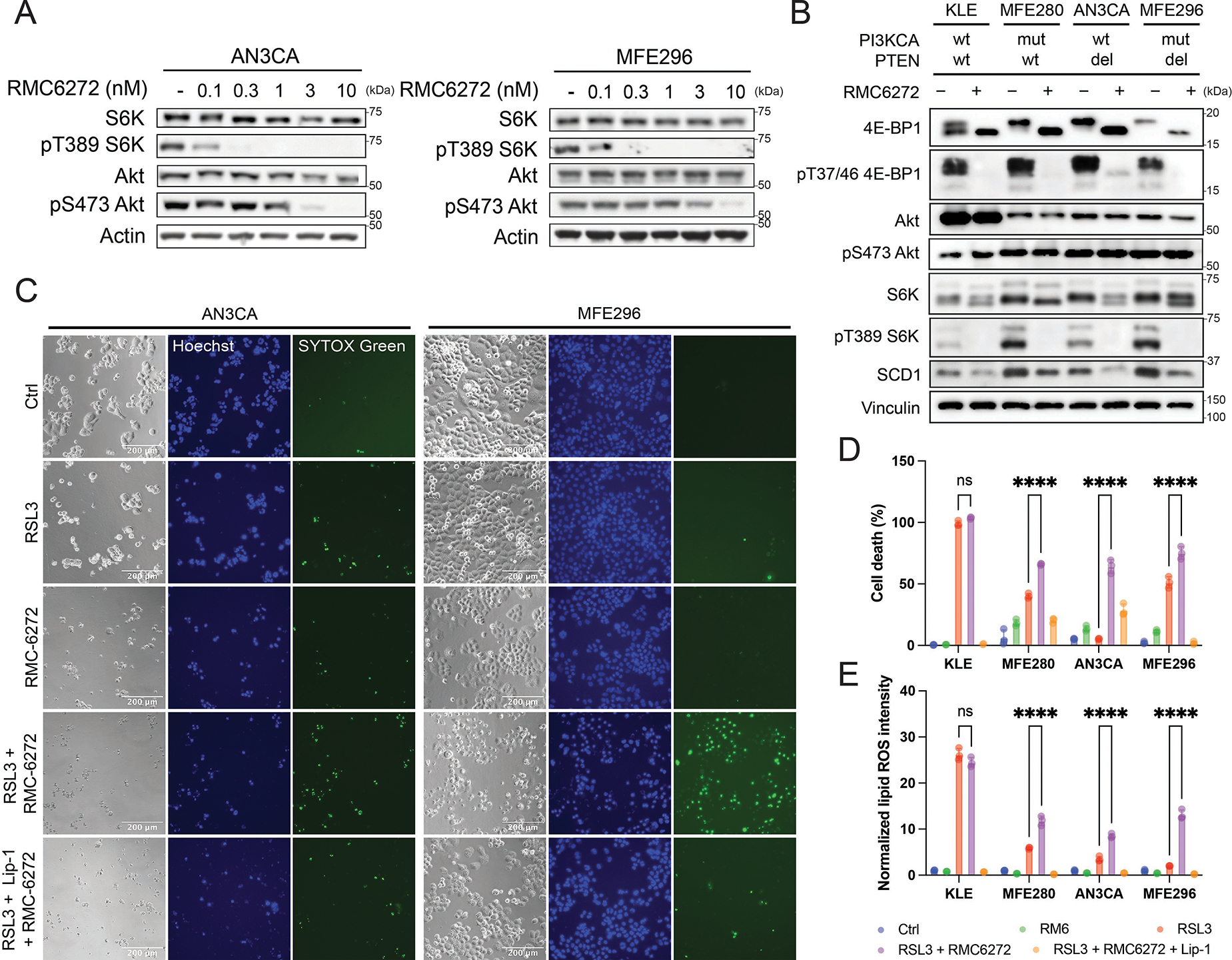
Bi-steric mTORC1 inhibitor RMC-6272 sensitizes EC cells to ferroptosis. (A, B) RMC-6272 inhibited mTORC1 activity in the nM range in AN3CA and MFE296 cells at 8 h; (C) Representative image of cell death at 24 h following 24 h treatment with RMC-6272 and subsequent RSL3 treatment; (D) Cell death at 24 h following 24 h treatment with RMC-6272 and subsequent RSL3 treatment on EC cell lines; (E) Normalized lipid ROS intensity of EC cell lines at 4 h following 24 h treatment with RMC-6272 and subsequent RSL3 treatment. RMC-6272, 1 nM; RSL3, 50 nM; Lip-1, 2 μM. Data are presented as mean ± SD, *n* = 3 (D-E) biologically independent samples. Statistical analysis was performed using two-way ANOVA (D-E). In all panels with combination treatment, when CCI-779 was used, cells were pretreated with CCI-779 for 24 h before the indicated combination treatment. mTORC1: mechanistic target of rapamycin complex 1; EC: endometrial cancer; PAM: PI3K-Akt-mTOR; ROS: reactive oxygen species; ANOVA: analysis of variance; SD: standard deviation.

To evaluate the efficacy of RMC-6272, we treated EC cell lines with varying concentrations of RMC-6272 (Figure 2A; Figure S2A,B). mTORC1 inhibition was evident at 0.1 nM, demonstrated by decreased phosphorylation of T389 S6K, and full inhibition was achieved at 0.3 nM. Consistent with this, treatment with 0.3 nM RMC-6272 was sufficient to sensitize PAM-activated EC cells to RSL3-induced ferroptosis (Figure S2C,D), confirming that selective mTORC1 inhibition is sufficient to enhance ferroptosis. At higher concentration in the nanomolar range, RMC-6272 began to inhibit mTORC2 as well, as measured by decreased phosphorylation of S473 Akt. To avoid mTORC2 inhibition, we used 1 nM RMC-6272 for subsequent experiments. Treatment of the four EC cell lines with 1 nM RMC-6272 confirmed complete shutdown of mTORC1 signaling, including inhibition of phosphorylated T389 S6K and T37/46 4E-BP1, with no inhibition of phosphorylated S473 Akt, indicating specificity towards mTORC1 at this dose (Figure 2B). Notably, this concentration is much lower than that required for CCI-779 to achieve a comparable level of inhibition, which is typically in the micromolar range.

To verify that mTORC1 inhibition by RMC-6272 sensitizes EC cells to ferroptosis induction, we pretreated cells with RMC-6272 and then induced ferroptosis with RSL3. As expected, PAM-activated EC cell lines showed significant sensitization to ferroptosis (Figure 2C,D,E; Figure S2E). This validates the combinational effect of PAM pathway inhibition with ferroptosis induction and supports the use of a low dose, potent bi-steric mTOR inhibitor for further therapeutic development.

We previously showed that mTORC1 inhibits ferroptosis through the mTORC1-SREBP1-SCD1 axis, where SCD1 converts SFAs into MUFAs, thereby inhibiting ferroptosis^[28]^. To test whether this mechanism applies in EC cells, we supplemented PAM-mutant cells with exogenous oleic acid (OA). Exogenous MUFA inhibited cell death induced by the combinational of RMC-6272 and RSL3 (Figure S2F). Furthermore, pharmacological inhibition of SCD1 using A939572 phenocopied mTORC1 inhibition by enhancing RSL3-induced cell death (Figure S2G). These results support that reduced SCD1 activity and MUFA availability contribute to ferroptosis sensitization upon mTORC1 inhibition.

### 3.4 mTORC1 inhibition induces apoptosis

**Figure 3.**
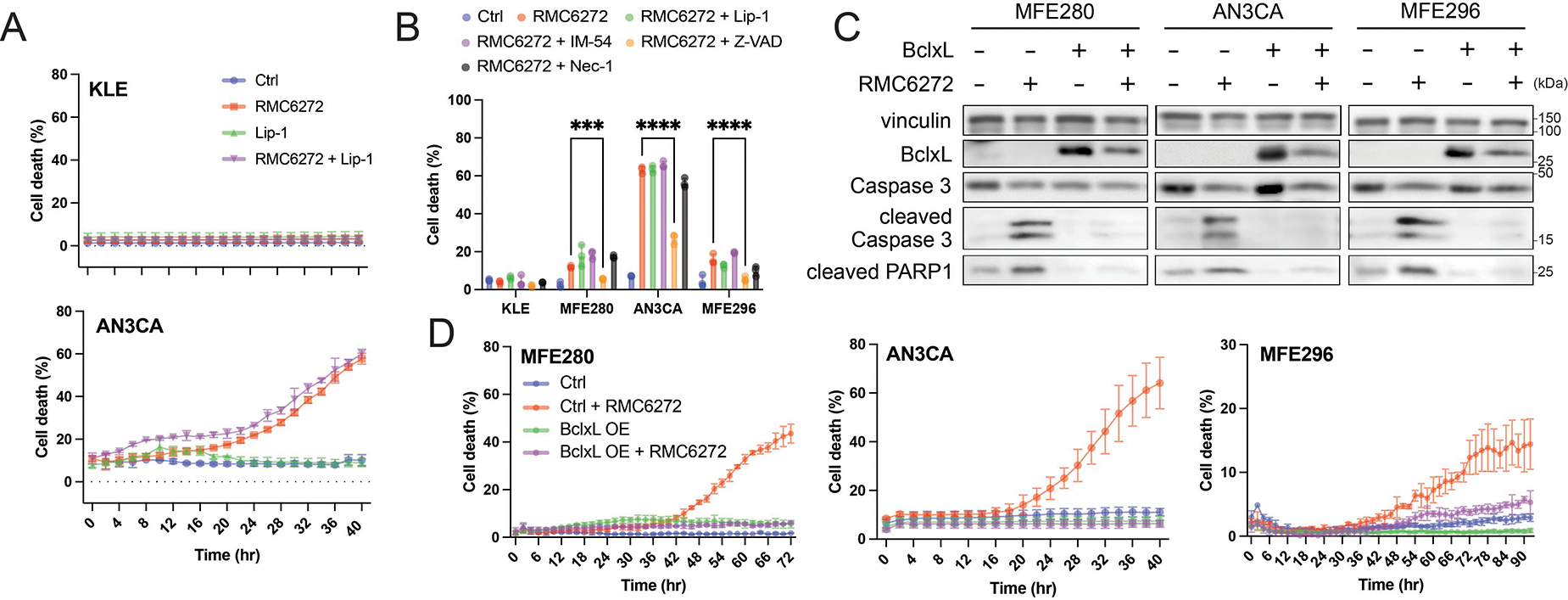
mTORC1 inhibition induces apoptosis in PAM pathway-mutated EC cells. (A) RMC-6272-induced cell death in EC cell lines with PAM pathway mutation, but not in the PAM-wildtype KLE cells; (B) RMC-6272-induced cell death was partially inhibited by apoptosis inhibitor Z-VAD-FMK at 54 h; (C) RMC-6272 upregulated cleavage of caspase 3 and PARP1 at 48 h post-treatment, and the cleavage was inhibited by Bcl-xL overexpression; (D) RMC-6272-induced cell death was inhibited by Bcl-xL overexpression. RMC-6272, 1 nM; Lip-1, 2 μM; IM-54, 10 μM; Z-VAD-FMK, 20 μM; Nec-1, 20 μM. Data are presented as mean ± SD, *n* = 3 (A, B, D) biologically independent samples. Statistical analysis was performed using two-way ANOVA (B). mTORC1: mechanistic target of rapamycin complex 1; EC: endometrial cancer; PAM: PI3K-Akt-mTOR; ANOVA: analysis of variance.

During our studies, we observed that upon longer treatment with RMC-6272, the PAM-mutant EC cells underwent cell death even in the absence of ferroptosis inducers (Figure 3A). Importantly, this cell death was not inhibited by ferroptosis inhibitor Liproxstatin-1 (Lip-1), suggesting a ferroptosis-independent mode of cell death. A similar response was observed in AN3CA cells treated with Torin for 24 hours, where cell death was also insensitive to Lip-1 (Figure 1C,D). This phenomenon was not observed in KLE cells, that lack PAM pathway activation (Figure 3A). To characterize this mode of cell death, we treated EC cells with RMC-6272 alongside various cell death pathway inhibitors. Of these, only the apoptosis inhibitor Z-VAD-FMK partially rescued cells from RMC-6272-induced cell death, indicating that apoptosis contributes at least in part to the observed cell death (Figure 3B). Consistently, RMC-6272-induced cell death was associated with cleavage of caspase-3 and PARP1, features of apoptosis. The overexpression of the anti-apoptotic protein Bcl-xL significantly reduced RMC-6272-induced cell death and the cleavage of caspase-3 and PARP1 (Figure 3C,D). Similarly, Z-VAD-FMK abolished Torin-induced cell death in AN3CA cells, confirming that apoptosis underlies this response as well (Figure S1E). Together, these data confirm that mTORC1 inhibition induces apoptosis in PAM-mutant EC cell lines, raising the possibility that combining mTORC1 inhibition with ferroptosis induction triggers multi-modal or combinatorial forms of cell death.

### 3.5 Combination of RMC-6272 and ferroptosis induction induces a multi-modal cell death *in vivo*

We next evaluated whether this same multi-modal cell death occurs *in vivo* upon the combination treatment. RMC-6272 alone has been reported to inhibit tumor growth in mice xenografts^[40]^, and we hypothesized that combining it with ferroptosis induction could further enhance therapeutic efficacy. To induce ferroptosis *in vivo*, we used JKE-1674, a GPX4 inhibitor suitable for animal studies^[41,42]^.

**Figure 4.**
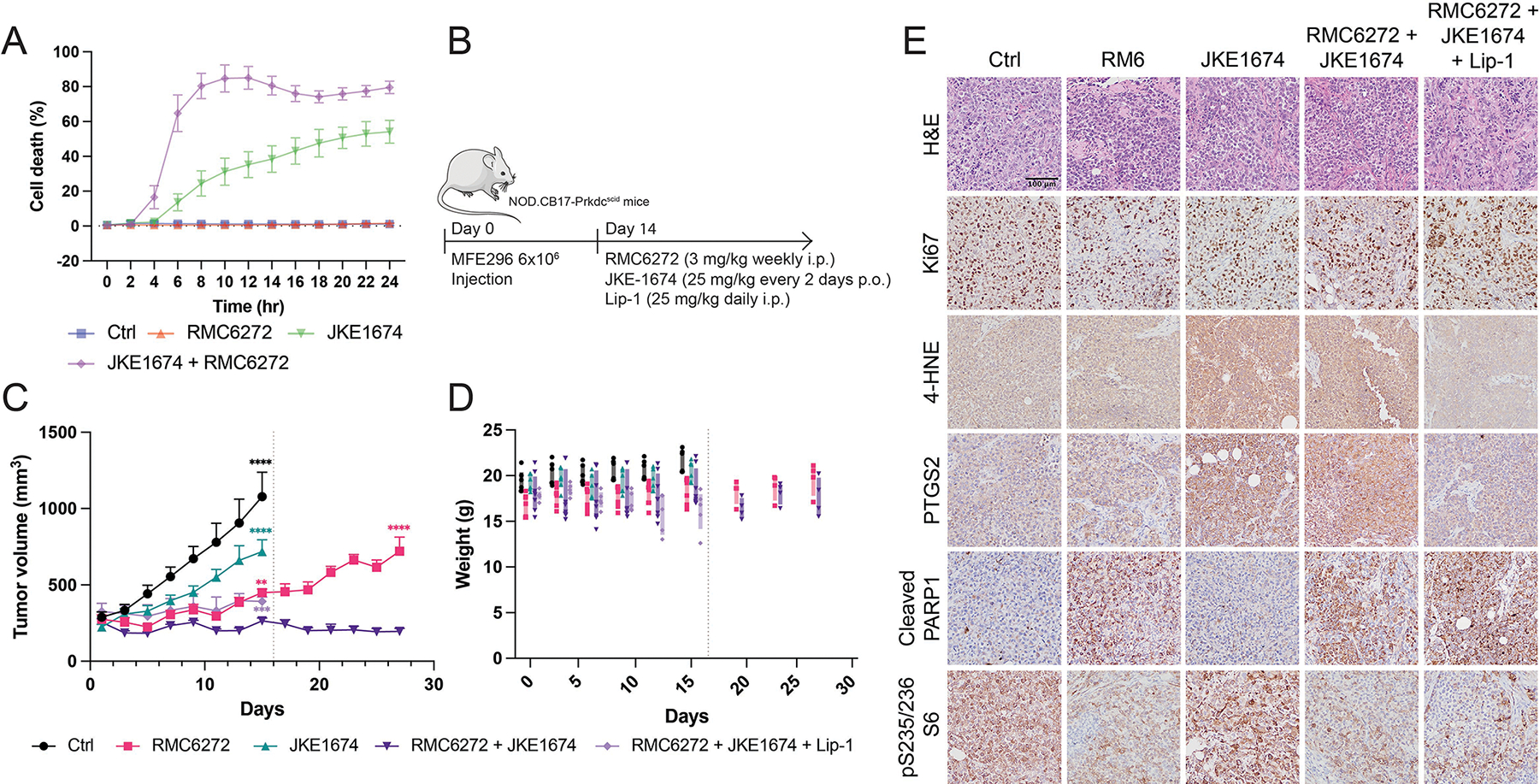
Combinational treatment of mTORC1 inhibition and ferroptosis induction leads to multi-modal cell death *in vivo*. (A) Cell death of the combination treatment of JKE-1674 with RMC-6272 in MFE296 cell line *in vitro*; (B) Experimental procedure of MFE296-tumor-bearing NOD.CB17-Prkdc^scid^ mice treated with RMC-6272, JKE-1674, and/or Lip-1 as indicated; (C) Tumor growth curves derived from MFE296 cells in xenograft mice model, starting at the day of drug treatment. Statistical analysis shows the comparison of RMC-6272 + JKE-1674 group with indicated groups at the indicated time point; (D) Weight of MFE296 tumor-bearing mice with indicated treatment, starting at the day of drug treatment; (E) Representative H & E and immunostaining images of Ki67, 4-HNE, PTGS2, cleaved PARP1, and phosphorylated S6 (S2235/236), from sections of xenograft tumors with indicated treatment. Data are presented as mean ± SD, *n* = 3 (A) or mean ± SEM, *n* as indicated in the methods section (C-D) for the number of biologically independent samples. Statistical analysis was performed using two-way ANOVA (C). mTORC1: mechanistic target of rapamycin complex 1; EC: endometrial cancer; PAM: PI3K-Akt-mTOR; ANOVA: analysis of variance; H & E: hematoxylin and eosin; SD: standard deviation; SEM: standard error of the mean.

We first confirmed the combinational effect with RMC-6272 *in vitro* (Figure 4A), and then MFE296 cells were injected subcutaneously into female NOD.CB17-Prkdc^scid^ mice to generate xenografts, followed by treatment with RMC-6272, JKE-1674, combination of both, or combination of both plus Lip-1 (Figure 4B). JKE-1674 alone produced mild tumor inhibition, RMC-6272 alone had a stronger effect, and the combination resulted in the most significant suppression of tumor growth, whereas Lip-1 mitigated the effect of the combination treatment toward that of RMC-6272 alone (Figure 4C). Drug-related toxicity was evaluated by monitoring body weight, and no significant weight loss was observed in these treatment groups during the experiment (Figure 4D). Remarkably, the pronounced therapeutic advantage of the combination over RMC-6272 alone was even more evident after a long period of treatment, when tumors appeared to become not responsive to RMC-6272 alone (Figure 4C).

Immunohistochemical (IHC) analysis of tumor sections demonstrated decreased phosphorylation of S235/236 residues in S6 protein in groups treated with RMC-6272, confirming mTORC1 inhibition. To assess mTORC2 activity in vivo, we examined Akt phosphorylation at S473 by IHC, which showed no observable change between RMC-6272 treatment group and the control group (Figure S3A). We also performed Western blot analysis on tumor lysates from three biological replicates per group. While phosphorylated Akt (S473) levels varied across samples, consistent with sample heterogeneity, there was no decrease in S473 pAkt upon RMC-6272 treatment (Figure S3B). These findings indicate that RMC-6272 selectively targets mTORC1 at the treatment concentration used in our mouse experiments. Apoptosis induction was evidenced by increased cleaved PARP1 in RMC-6272-treated tumors. Ferroptosis was detected by elevated 4-HNE and PTGS2 in JKE-1674-treated groups, which were inhibited by Lip-1 treatment (Figure 4E). Together, these findings establish that the combination therapy effectively induces both ferroptosis and apoptosis *in vivo*, implying a promising therapeutic approach for EC.

## 4. Discussion

In this study, we demonstrate that inhibition of mTORC1 in PAM pathway-activated EC sensitizes these cells to ferroptosis, thus providing the rationale for combining PAM pathway inhibitors with ferroptosis inducers for the treatment of PAM pathway-activated ECs.

Interestingly, EC cell lines with PAM pathway activation are generally resistant to ferroptosis induced by system x_c_^−^ inhibition (Figure S1B). Previous studies have shown that mTORC1 inhibition can promote ferroptosis through suppression of SREBP1/SCD1-mediated MUFA synthesis^[28]^, and that mTORC1 also regulates GPX4 synthesis through the Rag-mTORC1-4EBP pathway^[43]^. However, the specific PAM-pathway activated EC cells used in this study are resistant to system x_c_^−^ inhibition, with or without PAM pathway inhibition. The underlying mechanism for this resistance is not clear. Speculatively, this resistance could arise from PAM pathway-driven upregulation of cysteine synthesis via the transsulfuration pathway or alternative uptake mechanisms. For example, elevated level of cystathionine-β-synthase and cystathionine-γ-lyase, both components of the transsulfuration pathway, have been observed in Akt-hyperactivated cells^[44]^.

Notably, our data showed no clear difference in ferroptosis sensitivity between EC cells harboring single mutations in PTEN or PIK3CA and those with co-occurring mutations in both genes. The lack of observable difference might be due to that both PTEN loss and PIK3CA mutation hyperactivate mTOR signaling, such that either lesion alone suffices to confer ferroptosis resistance. Nonetheless, it is still highly plausible that the co-occurring mutations might provide certain yet-to-be-identified oncogenic or survival advantages to the tumor cells that either single mutation cannot.

Our study also revealed that mTORC1 inhibition by a bi-steric inhibitor induces apoptosis in PAM pathway-activated EC cells both *in vitro* and *in vivo*. The PAM pathway is linked to apoptosis regulation in several ways. For example, Akt can inhibit apoptosis through BAG phosphorylation^[45]^ or GSK3 phosphorylation^[46,47]^. Upregulation or overexpression of PTEN suppresses tumor cell growth and induces apoptosis^[48]^. It has also been reported that PI3K inhibition in combination with androgen receptor blockade induces apoptosis *in vivo*^[49]^. The absence of apoptosis in KLE cells following mTORC1 inhibition, in contrast to other PAM pathway-activated cell lines, may indicate PAM pathway-hyperactivated tumor becomes addicted to mTORC1 and relies on a strong mTORC1 activity for survival—it will be important and clinically relevant to define the underlying mechanisms.

While our study uncovers multi-modal cell death involving both ferroptosis and apoptosis upon the combinational treatment, there are several limitations that should be considered. First, many observations were derived from a limited panel of EC cell lines, which may not capture the full heterogeneity of PTEN and PIK3CA mutation contexts. The lack of detectable differences between single and double mutations could be influenced by cell line-specific biology or compensatory signaling and may not be generalizable to all tumors. Second, although apoptosis was observed following mTORC1 inhibition, the precise downstream mediators remain undefined, and may involve multiple overlapping mechanisms such as metabolic stress, Bcl-2 family regulation, or ER stress responses. Further studies will be needed to clarify these mechanisms and validate our findings in broader models. Nonetheless, our study sheds light on a potential therapeutic strategy for endometrial cancer by combining mTORC1 inhibition with ferroptosis induction, and it may inform patient stratification based on PAM pathway status while suggesting broader applicability of this approach to other cancers with hyperactivated mTOR signaling.

## 5. Conclusion

Our work demonstrates that the suppression of mTORC1 alters the vulnerability of EC cells with PAM pathway activation by promoting both ferroptotic and apoptotic cell death. The observation of this multi-modal cell death highlights a potential strategy to overcome resistance mechanisms and mitigate toxicity associated with current mTOR-targeting therapies. These findings warrant further investigation of selective mTORC1 inhibitors in combination with ferroptosis inducers, both in endometrial cancer and in other malignancies characterized by mTORC1 hyperactivation.

## Supplementary Material

supplementary materials

The supplementary material for this article is available at: Supplementary materials.

## Data Availability

The cancer patient datasets analyzed in this study were partially derived from the publicly accessible TCGA Research Network (The Cancer Genome Atlas), available at https://www.cancer.gov/tcga. Other materials and data could be obtained from the corresponding author upon reasonable request.
