# Supplementary material for: Targeting mTORC1 to promote ferroptosis and apoptosis in endometrial cancer with PI3K-Akt-mTOR pathway mutation
Source: Ferroptosis Oxid Stress. Author manuscript; Available in PMC 2025 Dec 27. (PMC12742288; doi:10.70401/fos.2025.0005)
Supplement: supplementary materials [file NIHMS2126070-supplement-supplementary_materials.pdf]

Ferroptosis and Oxidative Stress

https://doi.org/10.70401/fos.2025.0005

Supplementary information

Targeting mTORC1 to promote ferroptosis and apoptosis in endometrial cancer with PI3K-Akt-mTOR pathway mutation

Yingying Hu, Pei Liu, Neal Rosen, Xuejun Jiang

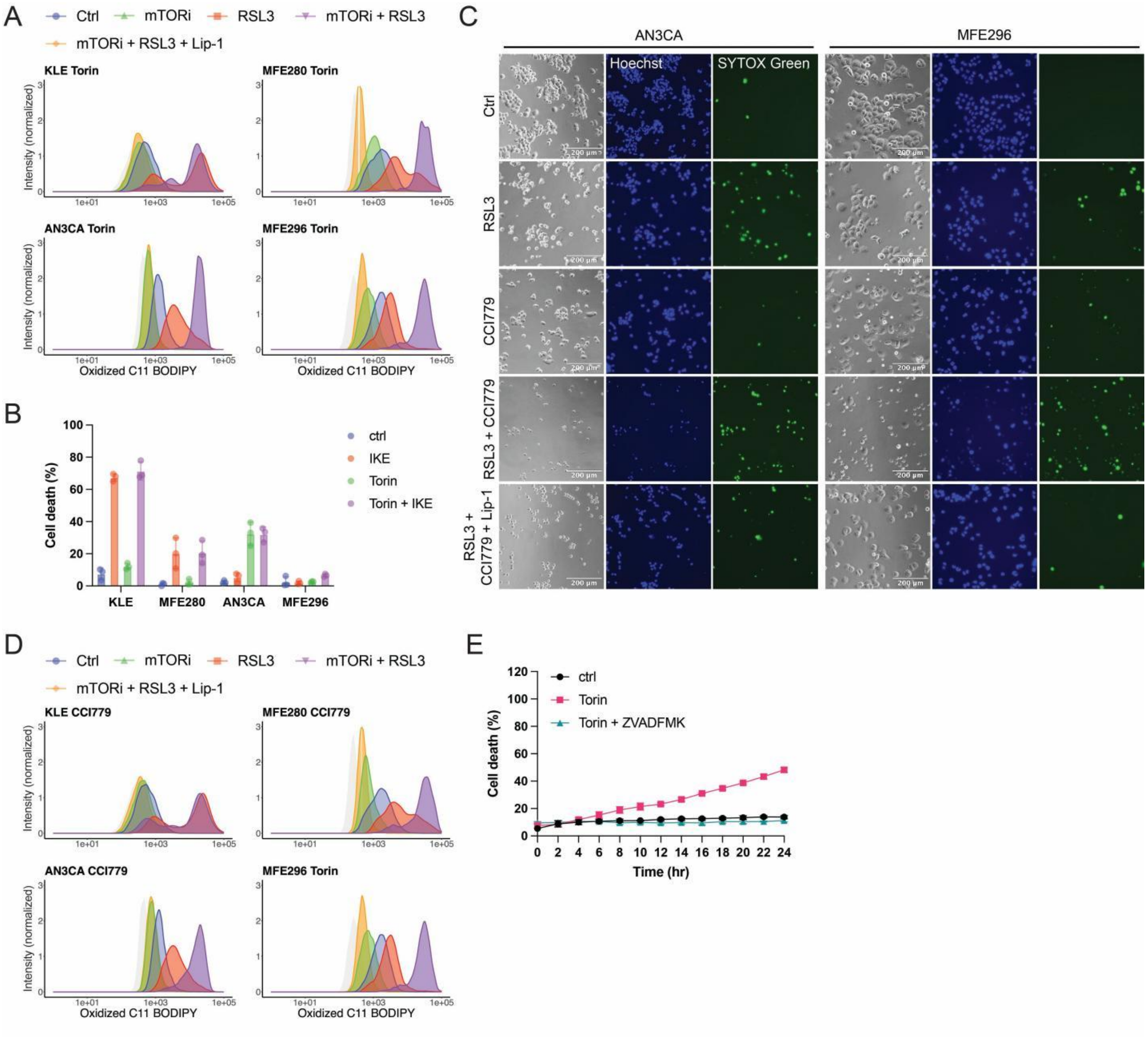

Figure S1. mTORC1 inhibition sensitizes PAM pathway-mutant EC cells towards ferroptosis. (A) Representative normalized lipid ROS intensity distribution by flow cytometry analysis after 4 hr of Torin and RSL3 treatment in EC cell lines; (B) Cell death at 36 hr post IKE treatment on EC cell lines; (C) Representative image of cell death at 24 hr following 24 hr treatment with CCI-779 and subsequent RSL3 treatment; (D) Representative normalized lipid ROS intensity distribution by flow cytometry analysis at 4 hr following 24 hr treatment with CCI-779 and subsequent RSL3 treatment in EC cell lines; (E) Torin-induced cell death is inhibited by Z-VAD-FMK. Torin, 1 μM; CCI-779, 2 μM; RSL3, 50 nM; Lip-1, 2 μM; IKE, 5 μM; Z-VAD-FMK 20 μM. Data are presented as mean ± SD, n = 3 (B,E). Statistical analysis was performed using two-way ANOVA (B). In all panels with combination treatment, when Torin or CCI-779 was used, cells were pretreated with Torin or CCI-779 for 24 hr before the indicated combination treatment.

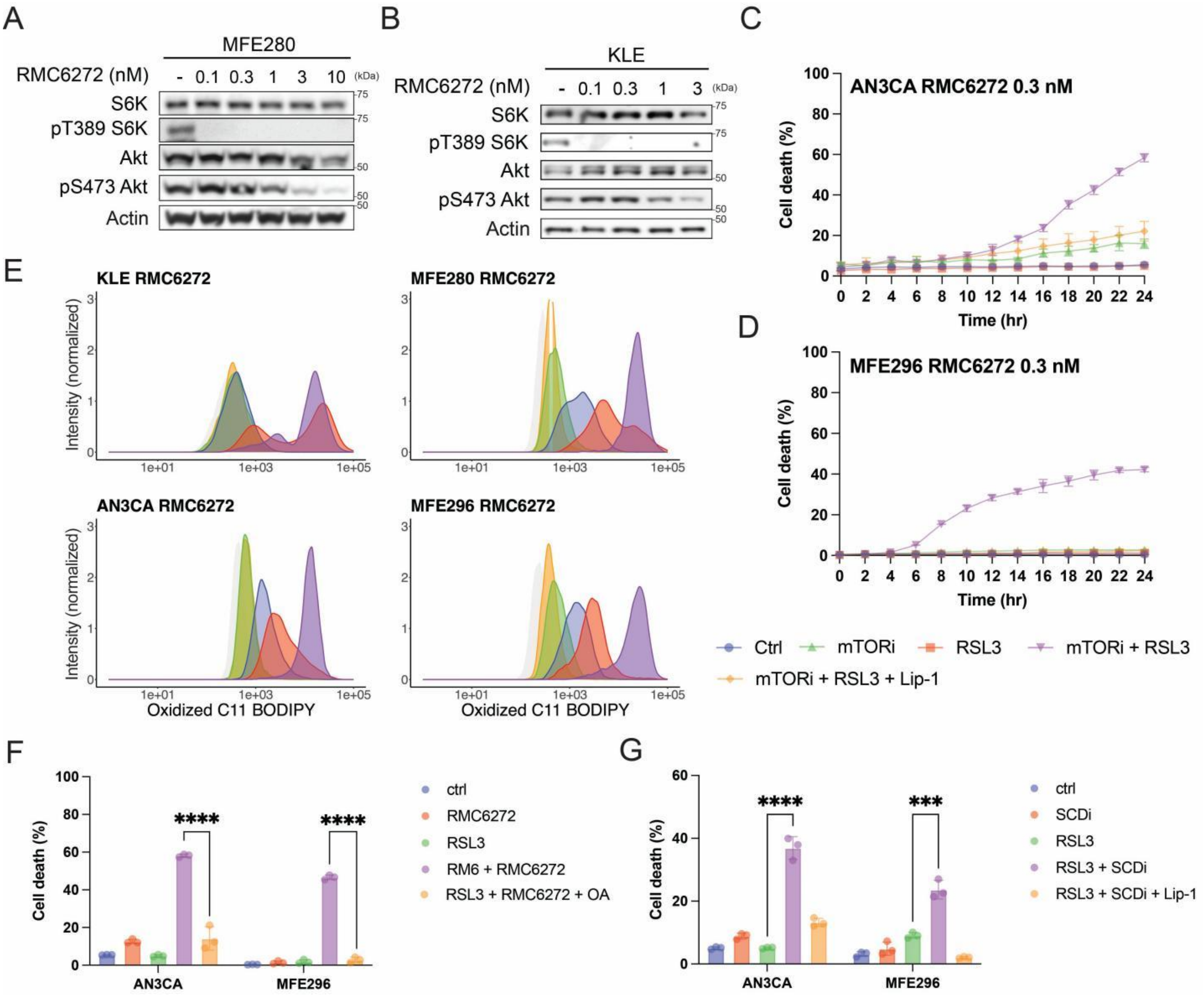

Figure S2. Bi-steric mTORC1 inhibitor RMC-6272 effectively sensitizes PAM pathway-mutant EC cells to ferroptosis. (A,B) RMC-6272 inhibits mTORC1 activity in the nM range in (A) MFE280 and (B) KLE cell lines within 8 hr treatment; (C,D) 0.3 nM RMC-6272 promotes RSL3-induced ferroptosis, RMC-6272 pretreatment for 24 hr, followed by 25 nM RSL3 treatment for another 24 hr in (C) AN3CA cells and (D) MFE296 cells; (E) Representative normalized lipid ROS intensity distribution by flow cytometry analysis following 24-hr treatment with RMC-6272 and subsequent RSL3 treatment for 4 hr in EC cell lines; (F) Oleic acid (OA) supplementation inhibits ferroptosis induced by 24 hr pretreatment of RMC-6272 and subsequent 25 nM RSL3 treatment; (G) Pharmacological inhibition of SCD1 using A939572 sensitized AN3CA and MFE296 cells to RSL3-induced at 28 hr following 24-hr pretreatment of A939572 and subsequent 25 nM RSL3 treatment. RMC-6272, 1 nM; RSL3, 50 nM; Lip-1 2 µM; A939572 20 µM, unless otherwise specified. Data are presented as mean ± SD, *n* = 3 (C,D, F,G) biologically independent samples. Statistical analysis was performed using two-way ANOVA (F,G). In all panels with combination treatment, when RMC-6272 was used, cells were pretreated with RMC-6272 for 24 hr.

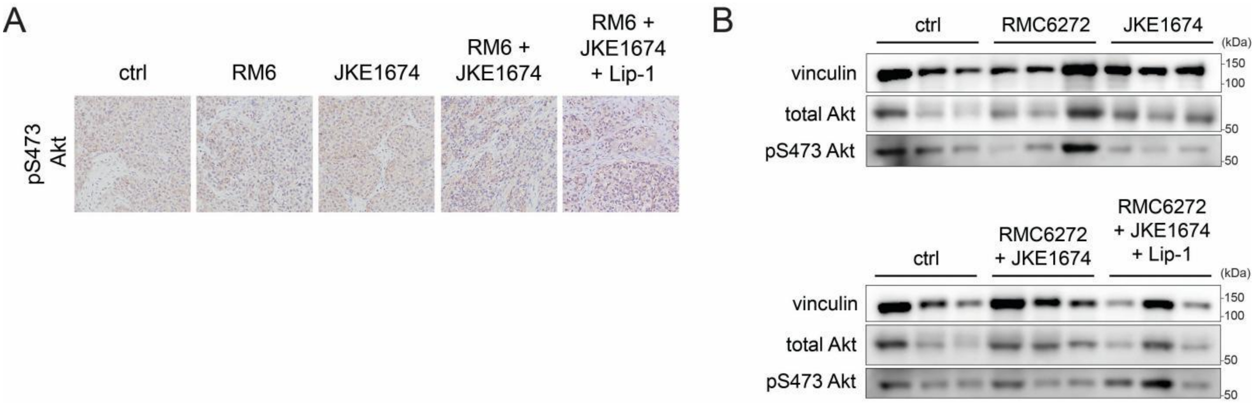

Figure S3. RMC-6272 selectively targets mTORC1 *in vivo*. (A,B) RMC-6272 does not inhibit Akt phosphorylation at S473, Western blot (A) and immunohistochemistry (B).
